# Diagnostic performance of novel inflammatory biomarkers based on ratios of laboratory indicators for nonalcoholic fatty liver disease

**DOI:** 10.3389/fendo.2022.981196

**Published:** 2022-11-28

**Authors:** Yanhua Zhao, Junxiang Xia, He He, Shanshan Liang, He Zhang, Wei Gan

**Affiliations:** ^1^ Department of Laboratory Medicine, West China Hospital, Sichuan University, Chengdu, China; ^2^ Department of Laboratory Medicine, Sichuan Province Orthopedic Hospital, Chengdu, China

**Keywords:** nonalcoholic fatty liver disease, monocyte to high-density lipoprotein cholesterol ratio, neutrophil to lymphocyte ratio, platelet to lymphocyte ratio, lymphocyte to monocyte ratio

## Abstract

**Introduction:**

There is few effective biomarkers for diagnosing nonalcoholic fatty liver disease (NAFLD) in clinical practice. This study was aimed to investigate the predictive ability of novel inflammatory biomarkers, including the monocyte to high-density lipoprotein cholesterol ratio (MHR), neutrophil to lymphocyte ratio (NLR), platelet to lymphocyte ratio (PLR), and lymphocyte to monocyte ratio (LMR), for NAFLD.

**Methods:**

A total of 4465 outpatients diagnosed with NAFLD and 3683 healthy controls were enrolled between May 2016 and November 2021 from the West China Hospital of Sichuan University, and anthropometric and laboratory examination data were collected. The two-sample Mann-Whitney U test and binary logistic regression analysis were used to evaluate the correlations between four inflammatory biomarkers and NAFLD. The areas under the curves (AUCs) of receiver operating characteristic were used to evaluate their predictive ability for NAFLD.

**Results:**

The MHR, NLR and LMR were higher in patients with NAFLD than in healthy controls (*P*<0.001), whereas the PLR was remarkably lower (*P*<0.001). The OR values of the MHR, NLR, PLR, and LMR were 1.599 (1.543-1.658), 1.250 (1.186-1.317), 0.987(0.986-0.988) and 1.111(1.083-1.139), respectively(*P*<0.001). After adjusting for confounding factors, MHR was still the most relevant risk factor for NAFLD compared with other inflammatory markers (*P*<0.001). The AUCs of the MHR, NLR, PLR, and LMR were as follows: 0.663 (0.651-0.675), 0.524 (0.512-0.537), 0.329 (0.318-0.341), and 0.543 (0.530-0.555), respectively (*P*<0.001). Furthermore, the diagnostic model combining the MHR with alanine aminotransferase, aspartate aminotransferase, total cholesterol, triglycerides, fasting blood glucose, creatinine, uric acid, and body mass index had the best AUC of 0.931 (0.925-0.936).

**Conclusions:**

MHR was superior to NLR, PLR and LMR as an inflammatory biomarker in the prediction of NAFLD. When combined with relevant laboratory parameters, the MHR may improve the clinical noninvasive diagnosis of NAFLD.

## Introduction

Nonalcoholic fatty liver disease (NAFLD), the most common chronic liver disease, is characterized by excessive hepatic triglyceride accumulation. The estimated global prevalence of NAFLD is 25% (1). According to the pathological process, NAFLD ranges from simple liver steatosis to nonalcoholic steatohepatitis (NASH), fibrosis and cirrhosis, eventually developing into end-stage liver disease, even hepatocellular carcinoma, causing a major health and economic burden for patients (2). Meanwhile, NAFLD is considered to have a strong bidirectional association with components of the metabolic syndrome, often accompanied by type 2 diabetes mellitus (T2DM), obesity, impaired lipid metabolism, and inflammation (3).Therefore, patients with NAFLD are not only at risk for liver-related complications, but are also associated with serious extrahepatic complications, such as adverse cardiovascular events and chronic kidney diseases (4), leading to a growing mortality and morbidity worldwide (5). With regard to metabolic events, an international panel of experts, including Eslam and George, recommended renaming NAFLD to metabolic associated fatty liver disease (MAFLD) in 2020 (6). Early diagnosis and intervention are of great significance to improve the prognosis of NAFLD patients.

Currently, the diagnosis of NAFLD requires imaging or liver biopsy (7). Although ultrasound, computed tomography and magnetic resonance spectroscopy (MRI) can be used for the qualitative diagnosis of NAFLD, their sensitivity is not ideal. Liver ultrasonography, a largely available procedure for fatty liver detection in clinical practice, can only detects steatosis when present in more than 20-30% of hepatocytes (8). Nevertheless, MRI can detect more than 5% of fatty changes, but it is expensive and difficult to popularize. As an invasive method, liver biopsy is often not accepted by patients, which not only causes bias in the diagnosis and severity assessment of the disease due to the small amount of sampling but also has many potential complications, such as bleeding, abdominal discomfort, pain, etc. (7) Although the new expert consensus affirms the value of blood biomarkers in the diagnosis of NAFLD (6), there is still a lack of effective biomarkers in clinical practice. Several steatosis biomarkers such as the hepatic steatosis index(HSI), fatty liver index(FLI) and NAFLD - liver fat score(LFS), have promising score for predicting NAFLD. However, these steatosis biomarkers have been validated against liver ultrasound or against MRI, and lack external validation (8). Consequently, the mining of NAFLD biomarkers has become an urgent clinical need.

In recent years, an increasing body of evidence has shown that chronic inflammation is considered to be a significant constituent of the pathophysiology of NAFLD (9, 10). It is possible to predict the existence and development of NAFLD by chronic inflammatory markers. Neutrophils, lymphocytes, monocytes and platelets play major roles in inflammatory processes. As indexes of systemic inflammation, the neutrophil to lymphocyte ratio (NLR), platelet to lymphocyte ratio (PLR) and lymphocyte to monocyte ratio (LMR) have been identified as useful biomarkers for the differential diagnosis or prognostic prediction of diseases, and even act as risk factors for obesity and metabolic syndrome (11–14). Most recently, considering the pro-inflammatory properties of monocytes and the anti-inflammatory properties of high-density lipoprotein (HDL), the ratio of monocyte to HDL-cholesterol (MHR) has been viewed as a novel systemic inflammatory marker used in clinical applications. Some studies show that MHR is associated with metabolic syndrome (15), acute coronary syndrome (16), and T2DM (17, 18). MHR has been shown to be an independent predictor of carotid artery disease in acute ischemic stroke (19) and diabetic retinopathy in T2DM patients (20). It is worth noting that the MHR can be used to assess MAFLD in patients with type 2 diabetes (21). However, there are few data showing the association between MHR and NAFLD, which is open to further exploration.

Therefore, this study aimed to investigate diagnostic performance of novel inflammatory biomarkers based on ratios of laboratory parameters, especially MHR, for NAFLD in a large case - control population and to evaluate the clinical value of inflammatory biomarkers combined with relevant laboratory indexes in the noninvasive diagnosis of NAFLD.

## Materials and methods

### Study population

Outpatients diagnosed with NAFLD at West China Hospital of Sichuan University from May 2016 to November 2021 were enrolled in this study. The diagnosis of NAFLD was based on the presence of fatty liver by histological examination or imaging techniques, excluding excessive alcoholic drinking (defined by an average daily alcohol consumption>20g) and other etiologies of chronic liver disease. The exclusion criteria were as follows: ①Patients with incomplete anthropometric parameters or laboratory test results; ②Patients who have been repeatedly submitted for examinations (only the data of the first visit were retained); ③Patients who had been receiving oral lipid-lowering drugs in the past two weeks; ④Patients receiving treatment with drugs known to promote liver steatosis (for example: tamoxifen, amiodarone, estrogen or corticosteroids); ⑤Patients receiving diabetes treatment drugs; ⑥Patients with malignant tumors or other severe organ dysfunction diseases; ⑦Women during pregnancy and lactation; ⑧Transplant patients. Meanwhile, apparently healthy individuals who were matched for age and sex to NAFLD patients during the same period for physical examination were included in the healthy control group. This study was approved by the Ethics Committee of West China Hospital of Sichuan University. All data were analyzed anonymously.

### Clinical and laboratory data collection

Anthropometric parameters, including height, weight, waist circumference (WC), hip circumference (HC), right arm systolic blood pressure (SBP), and diastolic blood pressure (DBP), were collected. Meanwhile, biochemical measurements of liver function, kidney function, fasting plasma glucose (FPG), and blood lipid contents as previously described were performed using an automatic biochemical analysis system (Roche Diagnostics GmbH, Mannheim, Germany) (22). Liver function tests included total bilirubin (TB), direct bilirubin (DB), alanine aminotransferase (ALT), aspartate aminotransferase (AST), alkaline phosphatase (ALP), *γ*-glutamate transpeptidase (GGT), total protein (TP), albumin (ALB). Serum enzymes included creatine kinase (CK), lactate dehydrogenase (LDH), and alpha-hydroxybutyrate dehydrogenase (HBDH). Kidney function tests included the determination of creatinine (CREA), uric acid (UA), urea and cystatin C (Cys-C) levels. Blood lipid tests were used to measure the concentrations of triglyceride (TG), total cholesterol (TC), high-density lipoprotein cholesterol (HDL-c), and low-density lipoprotein cholesterol (LDL-c). Additionally, peripheral blood cell counts, such as white blood cells, neutrophils, lymphocytes, monocytes and platelets were determined with a Sysmex XN-9100 automated blood cell analyzer (Sysmex, Kobe, Japan).

### Definition

MHR is calculated as the ratio of the absolute monocyte count divided by the HDL-c. The neutrophil to lymphocyte ratio (NLR), platelet to lymphocyte ratio (PLR) and lymphocyte to monocyte ratio (LMR) are the absolute value ratios of the corresponding blood cell counts. TG/HDL-c is the ratio of TG to HDL-c; residual cholesterol (RC) is equal to TC minus the sum of HDL-c and LDL-c; non-high density lipoprotein cholesterol (non-HDL-c) is equal to TC minus HDL-c. Body mass index (BMI) is calculated as weight divided by height squared, and waist-to-hip ratio (WHR) is the ratio of WC to HC.

### Statistical analysis

Statistical analysis was performed with SPSS version 26.0. Continuous variables were represented by the median (interquartile range), whereas categorical variables were described as the number of individuals and percentages. A two-sample Mann - Whitney U test was used to compare nonnormally distributed variables with nonparametric comparisons, and binary logistic regression analysis was used to evaluate the relationships between inflammatory biomarkers and NAFLD. The relationships between the MHR and other parameters were analyzed by Pearson’s correlation analysis. Receiver operating characteristic (ROC) curves were used to analyze the diagnostic efficacy of inflammatory biomarkers for NAFLD. *P* < 0.05 was considered statistically significant.

## Results

### Clinical, laboratory and inflammatory characteristics of the patients with NAFLD

**Figure 1:**
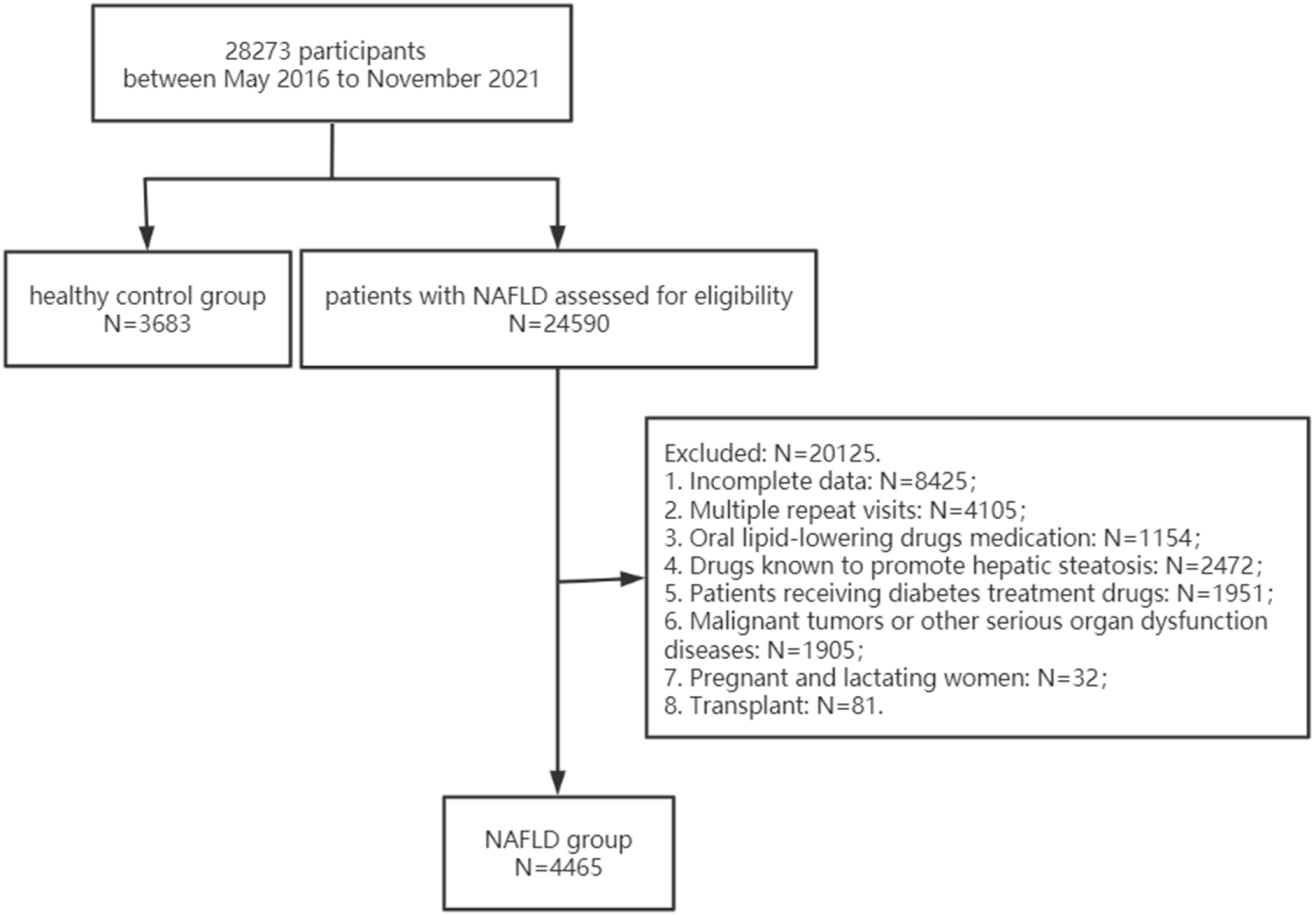
Flowchart describing the selection process of the study population.

Of the 24590 outpatients diagnosed with NAFLD from West China Hospital of Sichuan University between May 2016 and November 2021, 4465 patients (18.16%) were eventually included in this study. Of these, 13% of cases were defined by biopsy. Meanwhile, 3683 healthy individuals were enrolled in the control group (**Figure 1**). The anthropometric and laboratory parameters are shown in **Table 1**. Compared to the healthy controls, NAFLD patients had higher BMI indexes (*P*<0.001). With regard to laboratory parameters, the NAFLD group had a higher level of ALT, AST, ALP, GGT, LDH, HBDH, TBIL, FPG, TC, TG, UA, Cys-C, WBC, neutrophil, lymphocyte and monocyte counts but a lower level of HDL-c than the healthy group. To further investigate the inflammatory and lipid characteristics of the patients with NAFLD, novel inflammatory biomarkers and lipid parameters were evaluated. We found that inflammatory biomarkers, including the MHR, NLR and LMR, and lipid parameters, including non-HDL-c, RC and TG/HDL-c, were markedly higher in patients with NAFLD than in healthy controls (*P*<0.001), whereas the PLR was remarkably lower in the NAFLD group than in the controls (*P*<0.001).

**Table 1:** Comparison of clinical, laboratory and inflammatory characteristics between patients with NAFLD and healthy subjects.

Variables	NAFLD (N=4465)	Healthy controls (N=3683)	*P* value
Demographic parameters			
Age (year)	45 (36–53)	46 (37-52)	0.361
Male (n, %)	3236 (72.5)	2690 (73.0)	0.570
Smoking (n, %)	1081 (24.2)	840 (22.8)	0.138
Alcohol intake (n, %)	527 (11.8)	391 (10.6)	0.092
Anthropometric parameters			
BMI (kg/m^2^)	24.2 (21.6-26.7)	23.1 (21.3-24.7)	<0.001
WHR	0.84 (0.80-0.88)	0.82 (0.77-0.86)	<0.001
SBP (mmHg)	109 (102-121)	117 (110-124)	<0.001
DBP (mmHg)	69 (64-76)	71 (66-77)	0.003
Laboratory parameters			
TBIL (μmol/L)	13.3 (10.0-17.6)	12.4 (9.8-15.7)	<0.001
DBIL (μmol/L)	3.8 (2.9-5.2)	3.8 (3.0-4.7)	0.006
IBIL (μmol/L)	9.3 (6.9-12.5)	8.6 (6.8-11.1)	<0.001
TP (g/L)	75.4 (72.1-78.5)	75.5 (73.0-78.0)	0.068
ALB (g/L)	47.7 (45.4-49.8)	48.6 (46.8-50.3)	<0.001
GLB (g/L)	27.4 (24.8-30.4)	26.9 (24.7-29.1)	<0.001
TC (mmol/L)	4.67 (4.06-5.38)	4.63 (4.20-5.06)	<0.001
TG (mmol/L)	1.75 (1.23-2.54)	1.07 (0.83-1.36)	<0.001
HDL-c (mmol/L)	1.10 (0.92-1.32)	1.32 (1.14-1.55)	<0.001
LDL-c (mmol/L)	2.75 (2.21-3.30)	2.80 (2.42-3.19)	0.004
AST (IU/L)	29 (23-40)	21 (18-25)	<0.001
ALT (IU/L)	37 (24-60)	19 (14-26)	<0.001
ALP (IU/L)	81 (68-99)	73 (62-86)	<0.001
GGT (IU/L)	38 (23-72)	19 (14-27)	<0.001
CK (IU/L)	101 (75-138)	101 (80-133)	0.552
LDH (IU/L)	197 (170-228)	172 (156-190)	<0.001
HBDH (IU/L)	149 (129-173)	134 (122-148)	<0.001
FPG (mmol/L)	5.31 (4.88-5.93)	4.83 (4.56-5.11)	<0.001
CREA (μmol/L)	72 (61-83)	81 (72-89)	<0.001
UREA (mmol/L)	4.8 (4.0-5.8)	4.8 (4.1-5.5)	0.012
UA (μmol/L)	370 (312-435)	339 (288-388)	<0.001
Cys-C (mg/L)	0.86 (0.78-0.96)	0.82 (0.75-0.88)	<0.001
WBC (×10^9^/L)	6.30 (5.29-7.46)	5.54 (4.81-6.34)	<0.001
Platelet (×10^9^/L)	187 (145-234)	210 (176-244)	<0.001
Neutrophil (×10^9^/L)	3.63 (2.90-4.52)	3.15 (2.62-3.76)	<0.001
Lymphocyte (×10^9^/L)	1.99 (1.59-2.42)	1.77 (1.48-2.13)	<0.001
Monocyte (×10^9^/L)	0.39 (0.31-0.49)	0.37 (0.31-0.45)	<0.001
Novel lipid parameters			
Non-HDL-c (mmol/L)	3.53 (2.91-4.20)	3.26 (2.82-3.68)	<0.001
RC (×10^-2^ mmol/L)	6.8 (4.7-9.6)	4.4 (3.4-5.4)	<0.001
TG/HDL-c	1.56 (0.99-2.59)	0.81 (0.57-1.11)	<0.001
Novel inflammatory parameters			
MHR (×10^8^/mmol)	3.5 (2.6-4.8)	2.8 (2.1-3.6)	<0.001
NLR	1.82 (1.42-2.40)	1.77 (1.42-2.22)	<0.001
PLR	93.33 (72.03-120.25)	117.22 (94.02-142.11)	<0.001
LMR	5.09 (4.00-6.40)	4.83 (3.95-5.85)	<0.001

NAFLD, non-alcoholic fatty liver disease; BMI,body mass index; WHR, the ratio of waist circumference to hip circumference; SBP, systolic blood pressure; DBP, diastolic blood pressure; TBIL, total bilirubin; DBIL, direct bilirubin; IBIL, indirect bilirubin; TP, total protein; ALB, albumin; GLB, globulin; TC, total cholesterol; TG, triglycerides; HDL-c, high-density lipoprotein cholesterol; LDL-c, low-density lipoprotein cholesterol; ALT, alanine aminotransferase; AST, aspartate aminotransferase; ALP, alkaline phosphatase; GGT, γ-glutamyl transpeptidase; CK, creatine kinase; LDH, lactate dehydrogenase; HBDH, alpha-hydroxybutyrate dehydrogenase; FPG, fasting plasma glucose; CREA, creatinine; UREA, urea; UA, uric acid; Cys-C, cystatin-C; eGFR, estimated glomerular filtration rate; WBC, white blood cells; Non-HDL-c, non-high density lipoprotein cholesterol; RC, residual cholesterol; TG/HDL-c, triglycerides to high-density lipoprotein  ratio; MHR, monocyte to high-density lipoprotein cholesterol ratio; NLR, neutrophil to lymphocyte ratio; PLR, platelet to lymphocyte ratio; LMR, lymphocyte to monocyte ratio.

### Association between inflammatory markers and risk of NAFLD

**Figure 2:**
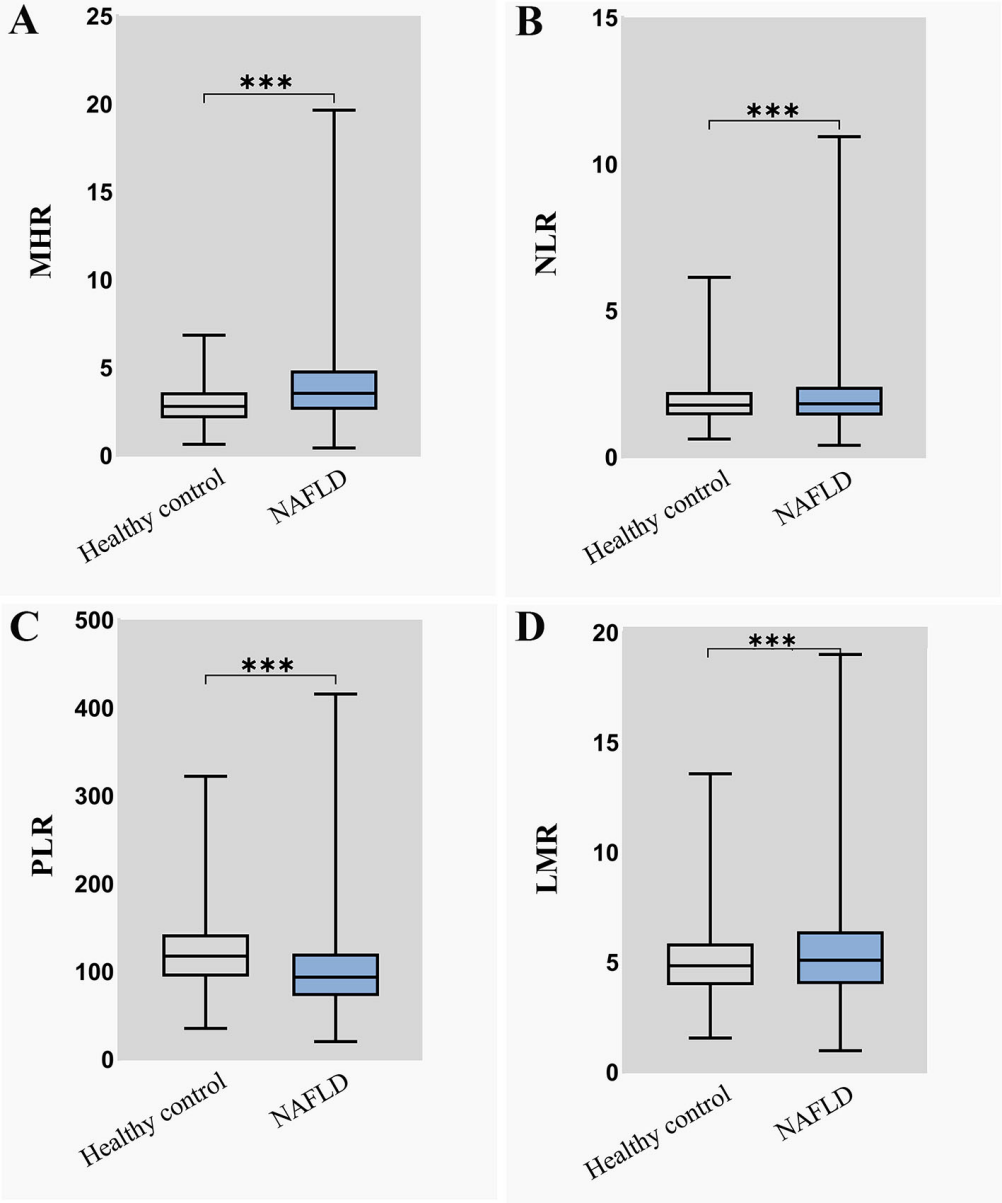
The distribution of four inflammatory markers in NAFLD group and healthy control group **(A)** MHR distribution in NAFLD group and healthy control group; **(B)** NLR distribution in NAFLD group and healthy control group; **(C)** PLR distribution in NAFLD group and healthy control group; **(D)** LMR distribution in NAFLD group and healthy control group. MHR, monocyte to high-density lipoprotein cholesterol ratio; NLR, neutrophil to lymphocyte ratio; PLR, platelet to lymphocyte ratio; LMR, lymphocyte to monocyte ratio.***means *P* < 0.001.

**Figure 3:**
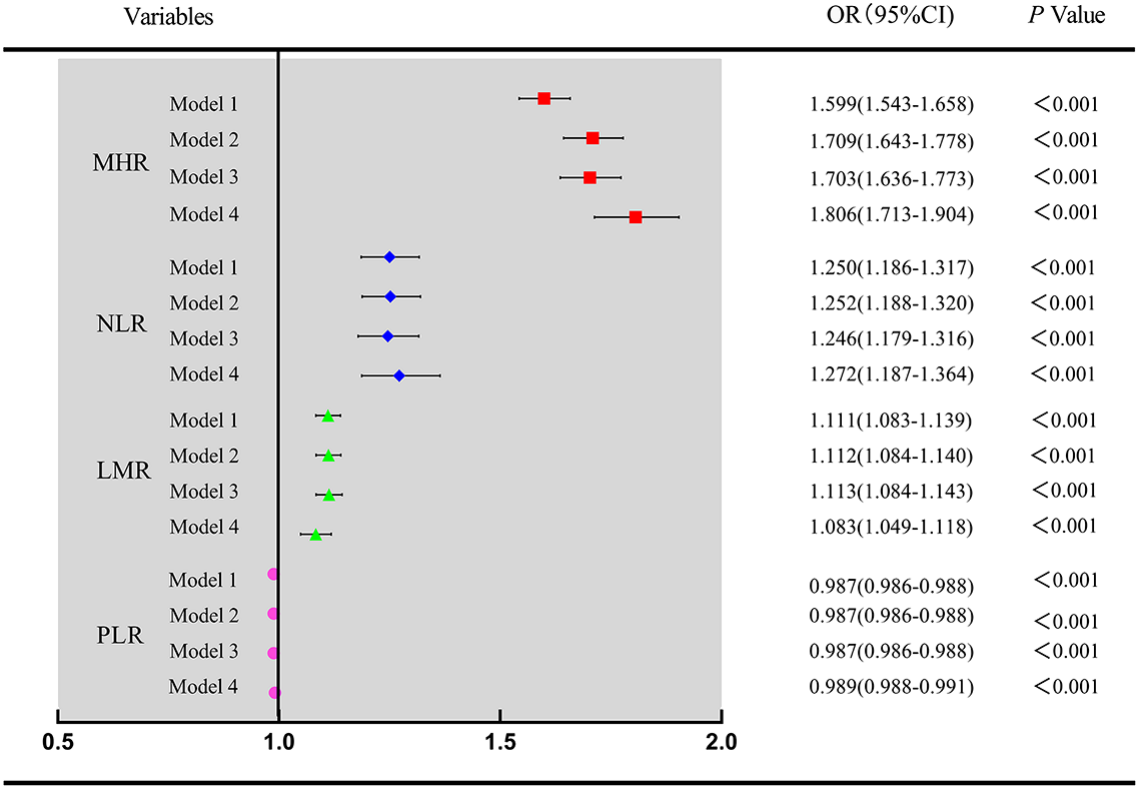
Univariate and multivariate analysis for four inflammatory markers associated with NAFLD Model 1 was unadjusted univariate analysis. Model 2 was adjusted for age, sex. Model 3 was further adjusted for BMI, SBP, DBP. Model 4 was further adjusted for FPG, TC, LDL-c, UA and CREA. MHR, monocyte to high-density lipoprotein cholesterol ratio; NLR, neutrophil to lymphocyte ratio; PLR, platelet to lymphocyte ratio; LMR, lymphocyte to monocyte ratio.

The distribution of the MHR, NLR, PLR, and LMR in the NAFLD group and healthy control group is illustrated in **Figure 2**. To explore the association between inflammatory markers and the risk of NAFLD, univariate and multivariate logistic regression were performed. As shown in **Figure 3**, the MHR, NLR, PLR, and LMR were independently associated with the risk of NAFLD in univariate and multivariate analyses, respectively. It is important to note that the MHR had a stronger relationship than the other biomarkers in the univariate analysis, as shown in model 1. After adjusting for sex and age in model 2, the OR value of the MHR was 1.709(1.643-1.778), which was still mostly correlated with NAFLD (*P*<0.001). After further adjusting for confounding factors, including BMI, SBP, DBP, FPG, TC, TG, LDL-c, UA and CREA, the OR value of MHR were 1.703(1.636-1.773) in model 3 (*P*<0.001) and 1.806 (1.713-1.904) in model 4 (*P*<0.001), respectively. Considering these confounding factors, MHR remained the most markedly associated factor with an increased risk for NAFLD compared with other inflammatory markers. In conclusion, MHR, NLR, PLR, and LMR were independent risk factors for NAFLD, while MHR was the most predominant risk factor.

### Correlations of MHR with relevant parameters

Pearson’s correlation analysis showed that the MHR was positively correlated with BMI, ALT, AST, TG, FPG, UA, CREA and TG/HDL-c (*P*<0.001), while it was negatively correlated with age, ALB, TC and LDL-c (*P*<0.001) (**Table 2**). These results suggest that there was a moderate correlation between the MHR and the associated metabolic profile.

**Table 2:** Relationships of MHR with relevant parameters.

Variable	r	*P* value
Age (years)	-0.117	<0.001
BMI (kg/m^2^)	0.108	<0.001
ALT(IU/L)	0.208	<0.001
AST(IU/L)	0.155	<0.001
ALB(g/L)	-0.196	<0.001
TG(mmol/L)	0.380	<0.001
TC(mmol/L)	-0.084	<0.001
LDL-c(mmol/L)	-0.087	<0.001
FPG(mmol/L)	0.160	<0.001
UA(μmol/L)	0.246	<0.001
CREA(μmol/L)	0.109	<0.001
TG/HDL-c	0.479	<0.001

BMI, body mass index; ALT, alanine aminotransferase; AST, aspartate aminotransferase; ALB, albumin; TG, triglycerides; TC, total cholesterol; LDL-c, low-density lipoprotein cholesterol; FPG, fasting plasma glucose; UA, uric acid; CREA, creatinine; TG/HDL-c, triglycerides to high-density lipoprotein  ratio.

### Evaluation of the diagnostic efficacy of inflammatory markers for NAFLD

**Figure 4:**
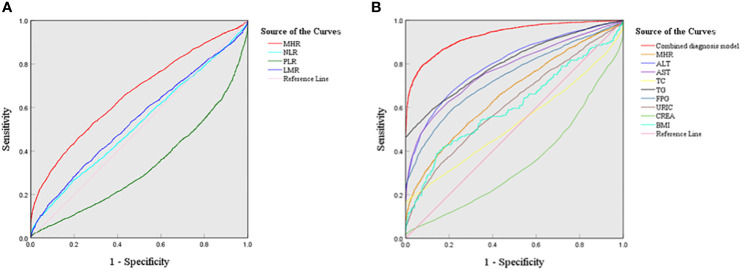
ROC curves of four inflammatory markers and the combined diagnostic model in NAFLD **(A)** ROC curves of four new inflammatory markers; **(B)** ROC curves of the combined diagnosis model.

To evaluate the predictive ability of inflammatory markers for NAFLD risk, ROC analysis was performed. As shown in **Figure 4A**, the AUCs of the four inflammatory markers were as follows: MHR 0.663 (95% CI: 0.651-0.675), NLR 0.524 (95% CI: 0.512-0.537), PLR 0.329 (95% CI: 0.318-0.341), and LMR 0.543 (95% CI: 0.530-0.555), respectively(*P*<0.001). This showed that the MHR had a higher predictive value for NAFLD than other indicators (*P*<0.001). The cutoff value of the MHR was 3.434 (×10^8^/mmol) with a sensitivity of 52.90% and a specificity of 71.20%.

To further evaluate the diagnostic performance of the MHR combined with serum indicators, the optimal diagnostic model was explored. As illustrated in **Figure 4B**, the combined diagnostic model combining the MHR with ALT, AST, TC, TG, FPG, CREA, UA, and BMI had the highest AUC value of 0.931 (95% CI: 0.925-0.936) with a sensitivity of 79.50% and a specificity of 92.10%.

## Discussion

The hepatic chronic inflammatory response is an important driving force of disease progression, as it promotes sustained hepatic fibrogenesis that contributes, in the setting of NAFLD, to the development of NASH and liver fibrosis (23). The triggers of liver inflammation in NAFLD can be systematic, including origins outside the liver (such as in adipose tissue and gut) and inside the organ (for example, lipotoxicity, innate immune response and cell death pathways) (9). Therefore, in this study, we investigated the correlation between systematic inflammatory biomarkers and NAFLD, especially MHR, a novel inflammatory marker, as well as its predictive value for NAFLD. The results demonstrated that the MHR value of NAFLD patients was significantly higher than that of healthy controls. It is worth noting that the MHR was better associated with the risk for NAFLD than the NLR, PLR and LMR.

In our research, the NAFLD group had a median age of 45 years, with 72.5% of males and 27.5% of females, which was consistent with that reported by Huang et al. (24). The study showed that the BMI of the NAFLD group was significantly higher than that of the healthy control group, consistent with Jia’s study (21), which suggested that overweight or obesity is a risk factor for NAFLD. With regard to biochemical parameters, liver function indexes including ALT, AST, ALP, GGT, LDH, TBIL, IBIL, and kidney function indexes contained UA and Cys-C, were significantly higher than those in healthy control subjects, although these median values were within the normal reference range. Meanwhile, compared to those in the healthy control group, patients with NAFLD had obviously higher plasma glucose levels and blood lipid content levels such as TC, TG, non-HDL-c, RC and TG/HDL-c, but lower HDL-c levels, which confirmed that NAFLD was closely associated with metabolic disorders. TG/HDL-c, a surrogate for insulin resistance, has been proven to better predict metabolic syndrome and NAFLD (25, 26).

NAFLD is a clinical syndrome characterized by hepatic steatosis, which can develop from simple steatosis to nonalcoholic steatohepatitis, and eventually to end-stage liver disease. It has become one of the main causes of cirrhosis and hepatocellular carcinoma (27). Additionally, NAFLD was independently and strongly associated with the risk for new heart failure through a number of potential mechanisms, particularly liver fibrosis (28). Following numerous studies, NAFLD was believed to be closely related to inflammation (9). Chronic liver inflammation induces carcinogenesis; it is often observed that patients with NAFLD eventually develop hepatocellular carcinoma (HCC) (29). The PLR, NLR and LMR have been reported to have inverse correlations with NAFLD, and are related to age, hyperuricemia, elevation of ALT, and low HDL-cholesterol (30, 31). Furthermore, using these markers is a simple and low-cost way to diagnose advanced liver fibrosis in NAFLD patients (32).

MHR, the ratio of monocyte to high-density lipoprotein cholesterol, was reported to be a novel marker of inflammation in recent years. Monocytes belong to the monocyte-phagocytic system (33). Monocytes are the first line of host defense, which are a key mediator of acute and chronic inflammation (34), and can induce immune-inflammatory responses (35). HDL-c has anti-inflammatory properties and may be essential for the prevention of other inflammatory diseases (36, 37). It has also been found that HDL can produce an anti-inflammatory response to macrophages under the mediating effect of cholesterol consumption (38). Elevated MHR was demonstrated to be associated with diabetic nephropathy (39). MHR may be a useful inflammatory marker for predicting metabolic syndrome patients and assessing disease severity (15, 40). However, studies on the association between the MHR and NAFLD are limited.

Our study demonstrated that the MHR had a higher value in patients with NAFLD than those in healthy control subjects. Using binary logistic analysis, MHR, NLR and MLR were all independent risk factors for NAFLD, while PLR was a protective factor for NAFLD in univariate analysis, as well as in multivariate analysis after adjusting for age, sex, BMI, SBP, DBP, FPG, TC, LDL-c, UA and CREA confounders. In another cross-sectional study, after adjusting for age, sex, BMI, waist circumference, SBP, DBP, ALT, TG, TC, FPG, and UA, the MHR was also still significantly connected with an increased risk of NAFLD (24). Notably, MHR remained the most markedly associated with an increased risk factor for NAFLD compared with the other inflammatory markers. Although sex, BMI and FPG were reported to influence the MHR index in metabolic subjects (41), the MHR was still superior to the NLR, PLR and LMR in the risk and protective factors for NAFLD after adjusting for these confounders in this study. Furthermore, the study showed that MHR was moderately correlated with age, BMI, ALT, AST, ALB, TC, TG, TG/HDL-c, LDL-c, FPG, UA and CREA.

To further explore the diagnostic value of the MHR in NAFLD, the clinical performance of the MHR, NLR, LMR and PLR was evaluated by the ROC curve analysis. Obviously, the AUC value of the MHR was the largest among the four novel inflammatory markers. The results of this study were consistent with Jia’s report based on the T2DM patients with metabolic associated fatty liver disease (21). Most importantly, our study provides a cut-off value of MHR of 3.434 (×10^8^/mmol) for NAFLD diagnosis. Through various combinations with relevant laboratory parameters, MHR combined with ALT, AST, TC, TG, FPG, CREA, URIC, and BMI may better predict NAFLD, with the best AUC value of 0.931. This may provide help for the noninvasive clinical diagnosis of NAFLD based on blood biomarkers. Furthermore, previous studies confirmed that HSI, FLI and NAFLD - LFS were accurate methods for diagnosing the presence of steatosis in NAFLD: AUROCs for HSI, FLI and LFS were 0.81, 0.80 and 0.83, respectively (8). The combined diagnostic model in our study had an AUC value of 0.931, which may have better performance than HSI, FLI and LFS for diagnosing steatosis in NAFLD.

Also, these indexes can be measured in clinical practice and are easier to get than HSI, FLI and LFS. In addition, literature data suggest that full-fledged cardiovascular events are likely predicted by blood components, which are reported to be associated with the presence/severity of NAFLD. A recent study demonstrated that hematocrit values was associated with early or subclinical atherosclerosis, in obese patients of various classes suffering from NAFLD (42). MHR, itself or in combination with other indicators, may also play an important role in predicting cardiovascular events of NAFLD. Further researches are needed to confirm these conclusions.

There are some limitations to our study. First, we did not perform subgroup analysis of the severity of NAFLD. Second, the influence of dietary habits was not evaluated in this study. Finally, our study is a cross-sectional study and cannot make a causal judgment on the relationship between MHR and NAFLD. Therefore, larger samples and multicenter studies are needed to further confirm the results of this research.

## Conclusions

MHR was superior to NLR, PLR and LMR as an inflammatory biomarker in the prediction of NAFLD. When combined with ALT, AST, TC, TG, FPG, CREA, UA, and BMI, the MHR may improve the clinical noninvasive diagnosis of NAFLD.

## Data availability statement

The raw data supporting the conclusions of this article will be made available by the authors, without undue reservation.

## Ethics statement

The studies involving human participants were reviewed and approved by the Ethics Committee of West China Hospital of Sichuan University. Written informed consent for participation was not required for this study in accordance with the national legislation and the institutional requirements.

## Author contributions

YZ was responsible for the study conception and design, and writing- original draft preparation. JX and HH were responsible for acquisition of data; SL and HZ were responsible for data analysis; WG was responsible for the conceptualization and visualization and the study, writing - reviewing and editing. All authors contributed to the article and approved the submitted version.

## Funding

This work was supported by the Sichuan Science and Technology Program (No.2021YFS0148, NO.2018SZ0382).

## Conflict of interest

The authors declare that the research was conducted in the absence of any commercial or financial relationships that could be construed as a potential conflict of interest.

## Publisher’s note

All claims expressed in this article are solely those of the authors and do not necessarily represent those of their affiliated organizations, or those of the publisher, the editors and the reviewers. Any product that may be evaluated in this article, or claim that may be made by its manufacturer, is not guaranteed or endorsed by the publisher.
